# Reframing the ozone–lung cancer link: toward exposure equity in urban China

**DOI:** 10.1016/j.jncc.2025.07.002

**Published:** 2025-08-05

**Authors:** Man Sun, Dan Zang, Jun Chen

**Affiliations:** Department of Oncology, The Second Hospital of Dalian Medical University, Dalian, China

To the editor,

We read with great interest the recent study by Wu et al.^1^ in the *Journal of the National Cancer Center*, which utilized a nationwide cohort of over two million Chinese adults to reveal a significant, nonlinear association between long-term ozone (O₃) exposure and lung cancer incidence. By extending the health implications of O₃ beyond cardiopulmonary effects to oncogenesis, the study offers timely evidence amid rising ground-level ozone concentrations and limited regulatory oversight. While the authors are to be commended for their rigorous methodology and thorough confounder adjustment, several scientific and socio-environmental dimensions merit further exploration.

First, the use of 10 km × 10 km township-level ozone estimates for individual exposure assessment may lead to non-differential misclassification. In a socio-spatially diverse country like China—where occupational patterns, commuting behavior, building insulation, and filtration access vary substantially—area-level averages can obscure behavior-modified ozone burdens in high-risk subgroups. Such spatial smoothing may dilute effect estimates and mask vulnerable populations.^2^ Given the long latency of lung carcinogenesis, future studies should adopt high-resolution exposure reconstruction—using wearable sensors, GPS-informed activity tracking, or land-use regression (LUR) models—to yield biologically relevant dose estimates and enable equity-sensitive risk prediction.^3^

Second, lung cancer outcomes were identified through linkage with the national cancer registry, yet key parameters—such as coverage, timeliness, and diagnostic accuracy—were not reported. Marked disparities in registry performance, particularly underreporting and delayed confirmation in rural or low-resource areas, may result in differential misclassification and compromise the validity of socioeconomic status (SES)- or region-stratified analyses.^4^ Future studies should include registry quality metrics and conduct sensitivity analyses to improve interpretability across spatial and demographic subgroups. Concurrently, enhancing audit protocols and implementing proactive case-finding strategies are essential to strengthen outcome reliability in environmental epidemiology and promote equity in China’s cancer surveillance infrastructure.

Paradoxically, the stronger ozone–lung cancer association among higher SES groups challenges the conventional assumption that environmental risks primarily burden the disadvantaged. Beyond disparities in diagnostic access, this may reflect structural exposure inequities inherent to urban design—where affluent areas are often situated in traffic-dense, heat-retaining zones with “urban canyon” effects that concentrate ozone precursors.^5^ Additionally, lifestyle patterns common among high-SES individuals—such as prolonged occupancy of sealed, air-conditioned spaces—may exacerbate microenvironmental ozone accumulation via limited ventilation and indoor–outdoor air recirculation. Together, these factors constitute a second-order environmental injustice: risk invisibility among “informed yet unprotected” populations. Addressing this requires ozone-sensitive spatial diagnostics, zoning reforms, and SES-adapted behavioral strategies that move beyond particulate-centric paradigms.

Wu et al.’s findings call for a paradigm shift in cancer prevention—redefining ozone as a frontline carcinogenic threat. As ambient ozone rises with China’s rapid urbanization, its integration into national cancer control strategies is both urgent and necessary.^6^ This requires updated exposure thresholds, climate-responsive urban design, and targeted protections for structurally vulnerable groups such as women, migrant workers, and high-SES residents in ozone-dense areas.^7^ Advancing exposure equity will further require a coordinated, multi-sectoral agenda. Policy instruments may include zoned ozone-day alerts, urban ventilation incentives, dynamic community-level risk platforms, and occupation-specific guidelines for outdoor labor. Collectively, these measures provide a strategic blueprint for equitable and resilient air carcinogen governance.

In summary, ozone can no longer remain peripheral in cancer prevention. This study underscores the urgency of aligning WHO carcinogenicity thresholds, improving China's exposure assessment infrastructure, and integrating ozone control into future-oriented urban cancer strategies.

## Declaration of competing interest

The authors declare that they have no known competing financial interests or personal relationships that could have appeared to influence the work reported in this paper.

**Author contributions**

M.S. conceived the study methodology, curated and visualized the data, and drafted the original manuscript. D.Z. contributed to the conceptualization of the study and critically revised the manuscript. J.C. provided supervision and contributed to the review and editing of the manuscript. All authors read and approved the final manuscript.
